# The ANGPTL4-HIF-1α loop: a critical regulator of renal interstitial fibrosis

**DOI:** 10.1186/s12967-024-05466-3

**Published:** 2024-07-11

**Authors:** Yan Li, Shuang Chen, Qian Yang, Xiao Liu, Weiming Zhou, Ting Kang, Weihua Wu, Santao Ou

**Affiliations:** 1 Department of Nephrology, The Affiliated Hospital of Southwest Medical University, 25 Taiping Street, Jiangyang District, Luzhou, 646000 Sichuan China; 2 Sichuan Clinical Research Center for Nephrology, Luzhou, 646000 Sichuan China; 3 Metabolic Vascular Disease Key Laboratory of Sichuan Province, Luzhou, 646000 Sichuan China

**Keywords:** Chronic kidney disease, Renal interstitial fibrosis, Angiogenin-like 4, Hypoxia-inducible factor-1α

## Abstract

**Background:**

Renal interstitial fibrosis (RIF) is a progressive, irreversible terminal kidney disease with a poor prognosis and high mortality. Angiopoietin-like 4 (ANGPTL4) is known to be associated with fibrosis in various organs, but its impact on the RIF process remains unclear. This study aimed to elucidate the role and underlying mechanisms of ANGPTL4 in the progression of RIF.

**Methods:**

In vivo, a chronic kidney disease (CKD) rat model of renal interstitial fibrosis was established via intragastric administration of adenine at different time points (4 and 6 weeks). Blood and urine samples were collected to assess renal function and 24-h urinary protein levels. Kidney tissues were subjected to HE and Masson staining for pathological observation. Immunohistochemistry and real-time quantitative PCR (qRT‒PCR) were performed to evaluate the expression of ANGPTL4 and hypoxia-inducible factor-1α (HIF-1α), followed by *Pearson* correlation analysis. Subsequently, kidney biopsy tissues from 11 CKD patients (6 with RIF and 5 without RIF) were subjected to immunohistochemical staining to validate the expression of ANGPTL4. In vitro, a fibrosis model of human renal tubular epithelial cells (HK2) was established through hypoxic stimulation. Subsequently, an HIF-1α inhibitor (2-MeOE2) was used, and ANGPTL4 was manipulated using siRNA or plasmid overexpression. Changes in ANGPTL4 and fibrosis markers were analyzed through Western blotting, qRT‒PCR, and immunofluorescence.

**Results:**

ANGPTL4 was significantly upregulated in the CKD rat model and was significantly positively correlated with renal injury markers, the fibrotic area, and HIF-1α. These results were confirmed by clinical samples, which showed a significant increase in the expression level of ANGPTL4 in CKD patients with RIF, which was positively correlated with HIF-1α. Further in vitro studies indicated that the expression of ANGPTL4 is regulated by HIF-1α, which in turn is subject to negative feedback regulation by ANGPTL4. Moreover, modulation of ANGPTL4 expression influences the progression of fibrosis in HK2 cells.

**Conclusion:**

Our findings indicate that ANGPTL4 is a key regulatory factor in renal fibrosis, forming a loop with HIF-1α, potentially serving as a novel therapeutic target for RIF.

## Introduction

Chronic kidney disease (CKD) has emerged as a significant public health concern worldwide, impacting 10–14% of the world's population [1, 2]. Renal interstitial fibrosis (RIF) represents the shared pathological alterations and pathways leading to the terminal stage of CKD progression [3]. The pathogenic mechanisms underlying RIF primarily involve hypoxia, inflammation, myofibroblast proliferation, epithelial–mesenchymal transition (EMT), excessive extracellular matrix (ECM) deposition, hemodynamic alterations, and energy metabolism changes. It constitutes a complex process involving diverse cellular elements, cytokines, and signaling pathways [4]. Currently, the etiology of RIF in CKD is intricate and has not been fully elucidated, and no effective preventive or therapeutic measures have been established. Consequently, elucidating the mechanisms governing the onset and progression of CKD-associated RIF, identifying novel targets, and developing innovative therapeutic modalities are highly important.

Angiopoietin-like 4 (ANGPTL4) belongs to the secreted protein superfamily of angiopoietin-like proteins. Structurally similar to angiopoietins, it does not bind to angiopoietin receptors but is typically considered an orphan ligand [8]. The primary structural elements of ANGPTL4 include the N-terminal signal peptide, N-terminal helical domain, connecting region, and C-terminal fibrinogen-like domain [9]. Functionally, ANGPTL4 plays a critical role in processes such as lipid metabolism, redox regulation, inflammation, angiogenesis, the hypoxic response, tissue damage repair, and tumor initiation and metastasis [10]. Its breadth of influence is intricately linked to its complex protein architecture. Currently, ANGPTL4 as a novel focal point for prognostic or predictive biomarkers across various malignancies and a burgeoning target in the realm of cancer therapies. For instance, recent studies have revealed that increased ANGPTL4 expression is closely associated with the invasiveness and drug resistance of KRAS^G12D^ pancreatic cancer [5, 6], and researchers have explored the mechanisms of drug resistance in KRAS-mutant cancers [7]. These findings may provide crucial molecular targets for new therapeutic strategies against KRAS-driven cancers.

Hypoxia is one of the pivotal events leading to RIF, and hypoxia-inducible factor-1α (HIF-1α) has emerged as the principal regulatory factor orchestrating the adaptive response to hypoxia in renal diseases [11, 12]. Previous investigations have elucidated the role of HIF-1α as an activator of ANGPTL4 [13], and HIF-1α promotes the progression of RIF [14–16]. However, the correlation between HIF-1α and ANGPTL4 in the context of CKD remains uncertain.

Recent studies have reported a close association between ANGPTL4 and pulmonary and hepatic fibrosis [17–19], potentially through mediating fibrogenesis through mechanisms such as inflammatory responses, excessive tissue repair, vascular genesis, and epithelial–mesenchymal transition [18]. Despite these findings, limited research has explored the role of ANGPTL4 in RIF. Our previous research preliminarily revealed the upregulation of ANGPTL4 expression in a CKD rat model [20]. However, substantive experimental validation is lacking.

Therefore, in this study, we investigated the role of ANGPTL4 in the pathogenesis of RIF by establishing in vivo and in vitro renal fibrosis models complemented by validation in human fibrotic kidney tissues.

## Methods

### Animal models

Male Sprague‒Dawley rats weighing between 170 and 180 g and aged 7 weeks were procured and maintained at the Animal Research Center of Southwest Medical University. The animals were acclimatized for one week prior to the experiment. Subsequently, the rats were randomly divided into a control group (n = 10) and a CKD group (n = 14). The CKD group was administered 2.5% adenine (Sigma‒Aldrich, St. Louis, Missouri, USA) via gavage at a dosage of 250 mg/kg [21]. The treatments were administered daily for the first 4 weeks and every other day from the 5th to the 6th week. The control group received an equivalent volume of physiological saline via gavage at the same time points. At the end of the 4th and 6th weeks postadministration, five rats from each group were randomly selected. After 24-h urine samples were collected using metabolic cages, the animals were euthanized, and kidney tissues and serum samples were collected. The “Guide for the Care and Use of Laboratory Animals” published by the National Institutes of Health in the United States served as the basis for the animal experimental protocol, which received approval from the Animal Ethics Committee of Southwest Medical University (SWMU20210397).

### Patients and samples

This study included a total of 11 patients with CKD who underwent renal biopsy at the Department of Nephrology, the Affiliated Hospital of Southwest Medical University, from January 2022 to December 2022. Based on the pathological findings from renal biopsies, the patients were stratified into the RIF group (n = 6) and the non-RIF group (n = 5). Among the 6 patients in the RIF group, 2 presented with focal proliferative sclerosing IgA nephropathy, 2 with mesangial proliferative glomerulonephritis, 1 with focal segmental glomerulosclerosis, and 1 with membranous nephropathy. The non-RIF group comprised 5 patients with minimal change disease. Renal tissue specimens were collected with the informed consent of the patients, and the study was approved by the Clinical Trial Ethics Committee of the Affiliated Hospital of Southwest Medical University.

### Cell culture

Professor Caiwei Qiu from the Affiliated Traditional Chinese Medicine Hospital of Southwest Medical University provided HK2 cells, which were cultured in DMEM/F-12 (C11330500BT, Gibco, Gaithersburg, USA) containing 10% fetal bovine serum (C04001, VivaCell, Shanghai, China). The cultivation conditions involved a carbon dioxide incubator set at 37 °C, 5% CO2, and 95% humidity, and the culture medium was changed every 2–3 days. Subsequently, the HK2 cells were incubated with 150 μmol/L Cocl_2_ (C805746; Macklin, Shanghai, China) for 24 h to induce cellular fibrosis [22]. To test this possibility, the HIF-1α inhibitor 2-MeOE2 (5 μmol/L) (S1233, Selleck, Texas, USA) [23], *ANGPTL4* small interfering RNA (siRNA), and overexpression plasmids (He Yuan Biotech, Shanghai, China) were used. The impact of these interventions on fibrosis in HK2 cells was then observed.

### Biochemical indices and renal histopathology

Serum creatinine (Scr), blood urea nitrogen (BUN), and 24-h urinary protein (24 h-Upro) levels were quantified using a fully automated biochemical analyzer (Sigma Chemical, Louis, Missouri, USA). Following established protocols, renal tissues were embedded in paraffin and subsequently processed into paraffin Sects. (4 μm). According to the respective instruction manuals, the kidney sections were deparaffinized, rehydrated, and baked at 65 °C before being subjected to staining procedures using a hematoxylin–eosin (HE) staining kit (C0105S, Beyotime, Shanghai, China) and a Masson trichrome staining kit (BA4079A, BASO, Zhuhai, China). Finally, morphological alterations in renal tissue and the extent of blue collagen fiber deposition were observed under a microscope (Nikon, Tokyo, Japan).

### Immunohistochemical staining

After deparaffinization and hydration, paraffin sections of renal tissue were subjected to antigen retrieval in Trisodium citrate buffer. Subsequent to the blockade of endogenous peroxidases, the sections were incubated overnight at 4 °C with antibodies targeting HIF-1α (1:100, 36,169, CST), ANGPTL4 (1:100, DF6751, Affinity), α-SMA (1:100, 19245S, CST), and Col-I (1:100, BA0325, Wuhan Boster). The following day, the sections were incubated at 37 °C with secondary antibodies (Zhongshan Golden Bridge, Beijing, China) for 20 min, followed by DAB chromogenic staining, hematoxylin nuclear counterstaining, dehydration, and slide mounting. Finally, observation and capture were performed using a microscope, and semiquantitative analysis was conducted using IPP 6.0 software.

### Quantitative real-time polymerase chain reaction (qRT–PCR)

Total RNA was extracted with TRIzol reagent, reverse transcribed into cDNA, and ultimately mixed with SYBR Green (all obtained from Vazyme Biotech, Nanjing, China). qRT‒PCR analysis was subsequently conducted on a LightCycler 480 II real-time polymerase chain reaction system (Roche, Basel, Switzerland). The primer sequences are detailed in Table 1. The final data were analyzed using the 2^^(−ΔΔCT)^ method, and the mRNA expression levels were normalized to those of the *β-actin* gene.

**Table 1:** qRT‒PCR primer sequences

Gene	Species	Forward primers (5'-3')	Reverse primers (5'-3')
*Hif-1α*	Rat	CTCCCTTTTTCAAGCAGCAG	GCTCCATTCCATCCTGTTCA
*Angptl4*	Rat	AAGAGGCTTCCCAAGATGGC	GAAGTCCACAGAGCCGTTCA
*α-sma*	Rat	GCGTGGCTATTCCTTCGTGACTAC	CCATCAGGCAGTTCGTAGCTCTTC
*Col-I*	Rat	GGATCGACCCTAACCAAGCC	GATCGGAACCTTCGCTTGCA
*β-actin*	Rat	CCCATCTATGAGGGTTACGC	TTTAATGTCACGCACGATTTC
*HIF-1α*	Human	GAACGTCGAAAAGAAAAGTCTCG	CCTTATCAAGATGCGAACTCACA
*ANGPTL4*	Human	GTCCACCGACCTCCCGTTA	CCTCATGGTCTAGGTGCTTGT
*α-SMA*	Human	AAAAGACAGCTACGTGGGTGA	GCCATGTTCTATCGGGTACTTC
*COL-I*	Human	GAGGGCCAAGACGAAGACATC	CAGATCACGTCATCGCACAAC
*β-ACTIN*	Human	CATGTACGTTGCTATCCAGGC	CTCCTTAATGTCACGCACGAT

### Western blotting assays

Protein extraction and quantification were conducted using RIPA lysis buffer containing proteinase and phosphatase inhibitors and a BCA protein assay kit (P0010, Beyotime, Shanghai, China). Equivalent amounts of total protein were subjected to electrophoresis (8–10% SDS‒PAGE) and subsequently transferred onto a polyvinylidene difluoride membrane. The membrane was blocked with 5% nonfat milk for 1 h, followed by overnight incubation at 4 °C with the following primary antibodies: anti-HIF-1α (1:1000), anti-ANGPTL4 (1:500), anti-α-SMA (1:1000), anti-Col-I (1:500), and anti-β‐actin (1:5000) (66,009–1-lg, Wuhan, Proteintech). Subsequently, the membrane was incubated at room temperature for 1 h with secondary antibodies conjugated with horseradish peroxidase (1:5000) (Beyotime, Shanghai, China). Finally, the protein bands were visualized and quantified using enhanced chemiluminescence solution (WBKLS0100; Merck Millipore, Darmstadt, Germany) and ImageJ software.

### Immunofluorescence staining

HK2 cells were inoculated into cell-climbing slices. Following the prescribed procedures, the cells were washed three times with PBS, sequentially fixed with a 4% polyformaldehyde solution, permeabilized with 0.2% Triton-X100, and blocked with 10% goat serum. Subsequently, the sections were incubated overnight at 4 °C with primary antibodies against HIF-1α (1:500) and ANGPTL4 (1:50). On the following day, the cells were incubated with a fluorescently labeled secondary antibody (1:800) (HA1121, Huaan, Hangzhou, China) at room temperature in the absence of light for 1 h. Nuclear staining with DAPI and mounting were subsequently performed. Finally, the samples were observed and imaged using a fluorescence microscope (Nikon, Tokyo, Japan).

### Cell transfection

*ANGPTL4*-siRNA, pcDNA3.1( +)-ANGPTL4, and their corresponding negative controls were obtained from HeYuan Biotech (Shanghai, China). HK2 cells were cultured in six-well plates and transfected when they reached 80% confluence. For the transfection experiments, Lipofectamine 3000 (L3000015; Invitrogen, Carlsbad, California, USA) reagent was used at a dose of 100 nM siRNA per well or 2.5 g of plasmid per well for a 24-h transfection period, after which the cells were induced with cobalt chloride (Cocl_2_) for 24 h. The target sequences for *ANGPTL4* gene siRNAs were as follows: forward primer, 5'-GAACAGCAGGAUCCAGCAACUTT-3', and reverse primer, 5'-AGUUGCUGGAUCCUGCUGUUCTT-3'.

### Statistical analysis

All the data were statistically analyzed using GraphPad Prism 8.0 software (GraphPad Software, San Diego, California, USA). Descriptive statistics for the quantitative variables are presented as the mean ± standard deviation. Independent t tests were used for between-group parameter comparisons, while one-way analysis of variance (ANOVA) was used for multiple group comparisons. *Pearson* correlation analysis was performed to assess the correlation between two indicators. A *P* < 0.05 was considered to indicate statistical significance.

## Results

### Establishment of the CKD rat model

**Fig. 1:**
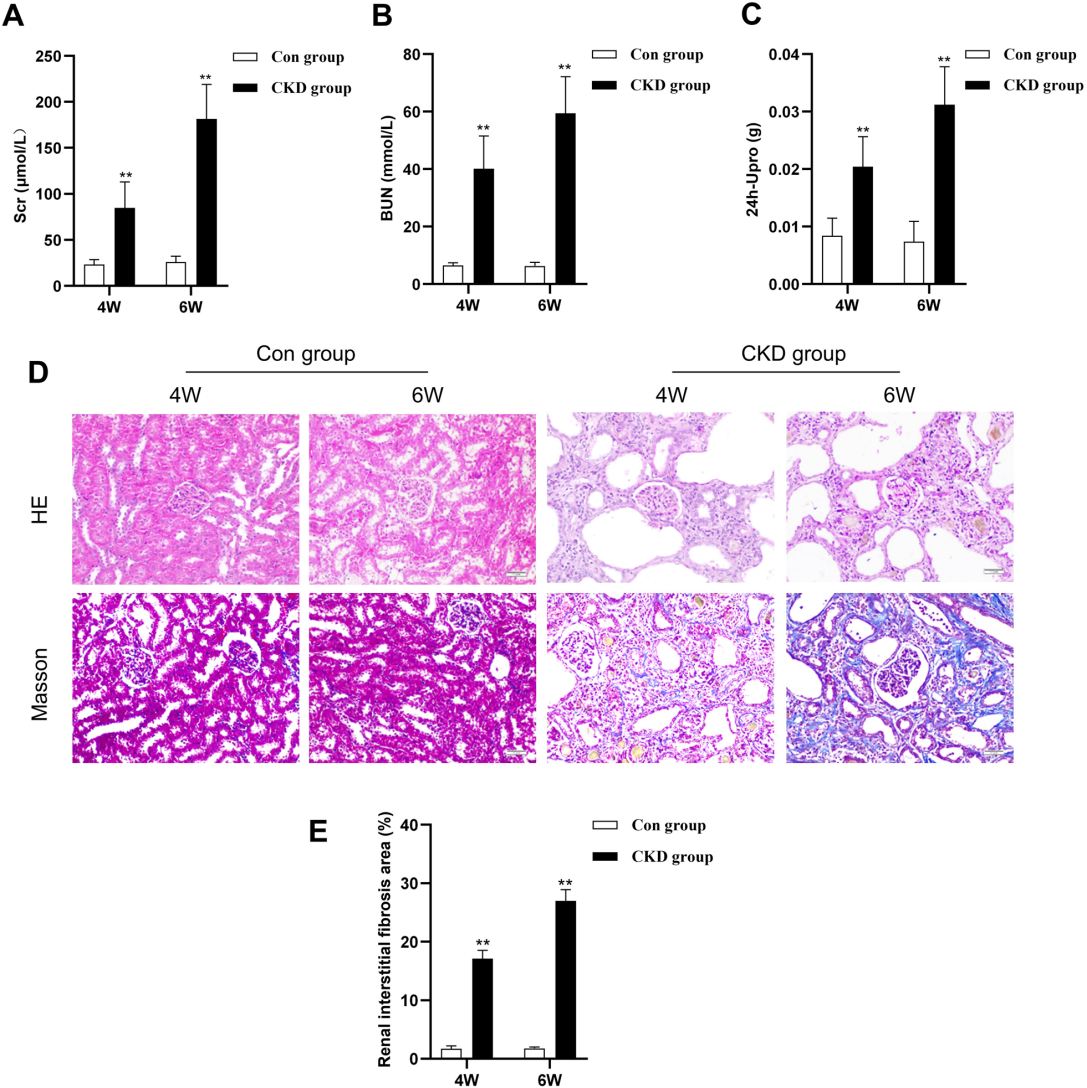
Establishment of the CKD rat model. The rats in the control group and the CKD group after 4 and 6 weeks of adenine gastric infusion were compared. **A**–**C** Quantities of Scr, BUN, and 24 h-Upro (n = 5/group). **D** HE staining of renal tissue. Masson staining revealed the deposition of blue collagen fibers (× 200 magnification, scale bar = 50 µm). **E** ImageJ was used for the quantitative assessment of the renal interstitial fibrosis area. All the data are presented as the mean ± standard deviation; *P < 0.05, **P < 0.01 vs. the control group at the same time

We administered 2.5% adenine (250 mg/kg) through gastric gavage to SD rats for 4 or 6 weeks to establish the CKD model. Initially, we assessed renal function. The CKD group had significantly greater levels of serum creatinine (Scr), blood urea nitrogen (BUN), and 24-h urinary protein (24 h-Upro) than did the control group (Fig. 1A–C), and these levels continued to increase over time. Subsequently, renal pathological changes were evaluated using HE and Masson staining. HE staining revealed that the renal tissues of the control group exhibited normal structural organization, while those of the CKD group exhibited structural disarray. These changes included a decrease in glomerular size and atrophy, clear dilation of renal tubules with luminal destruction, inflammatory cell infiltration, and the deposition of brownish-yellow particles (Fig. 1D). Furthermore, Masson staining of the CKD group demonstrated a substantial accumulation of blue collagen fibers in the glomeruli and renal interstitium but not in the control group (Fig. 1D). Compared with that in the control group, the fibrotic area in the kidneys in the CKD group was significantly greater at various time points (Fig. 1E).

### Increased expression of HIF-1α and ANGPTL4 in CKD rats

**Fig. 2:**
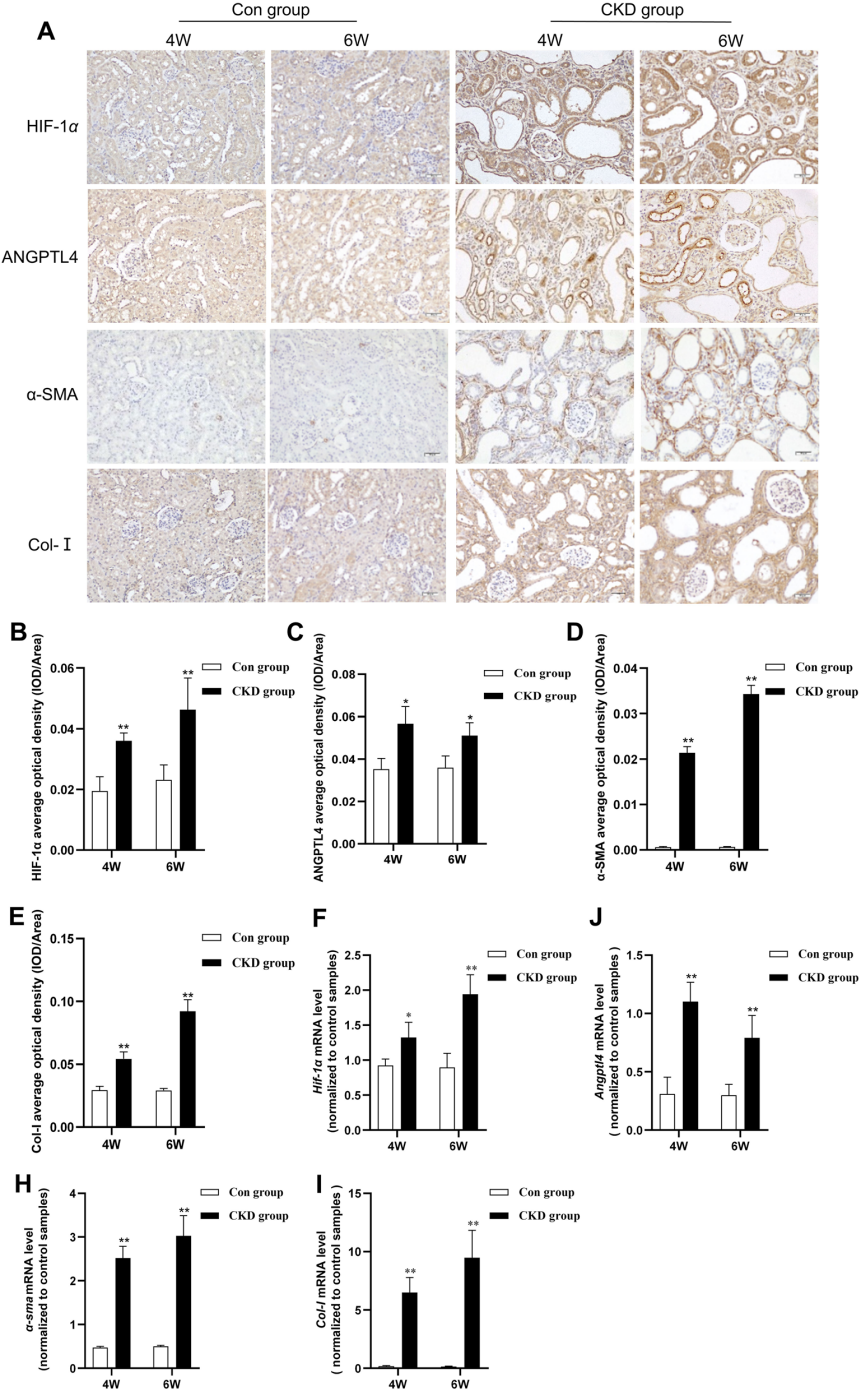
Increased expression of HIF-1α and ANGPTL4 in CKD rats. **A** Immunohistochemical staining to assess the expression of HIF-1α, ANGPTL4, α-SMA, and Col-I in the renal tissues of the two groups (× 200 magnification, scale bar = 50 µm). **B**–**E** Quantification of immunohistochemical staining (n = 5/group). **F**–**I** qRT‒PCR analysis of the expression of *Hif-1α*, *Angptl4*, *α-sma*, and *Col-I* mRNA in the renal tissues of the two groups of rats. All the data are presented as the mean ± standard deviation; *P < 0.05, **P < 0.01 vs. the corresponding control group at the same time point

Immunohistochemical staining revealed decreased HIF-1α and ANGPTL4 protein expression in the control group, whereas at various time points, there was a significant increase in HIF-1α and ANGPTL4 expression in the CKD group (Fig. 2A–C). The majority of the genes are expressed in proximal renal tubules, with modest expression in renal glomeruli. Compared to those in the control group, the expression of the alpha-smooth muscle actin (α-SMA) and collagen type I (Col-I) proteins in the renal interstitium in the CKD group increased at various time points (Fig. 2D, E). These proteins are considered indicative of interstitial or fibrotic phenotypes. Similarly, the qRT − PCR results demonstrated that the mRNA levels of *Hif-1α*, *Angptl4*, *α-sma*, and *Col-I* in the renal tissues of CKD rats at various time points were consistently greater than those in the control group (Fig. 2F–I). The findings of this study revealed a synchronous increase in the expression of HIF-1α and ANGPTL4 with the progression of RIF in CKD rats.

**Fig. 3:**
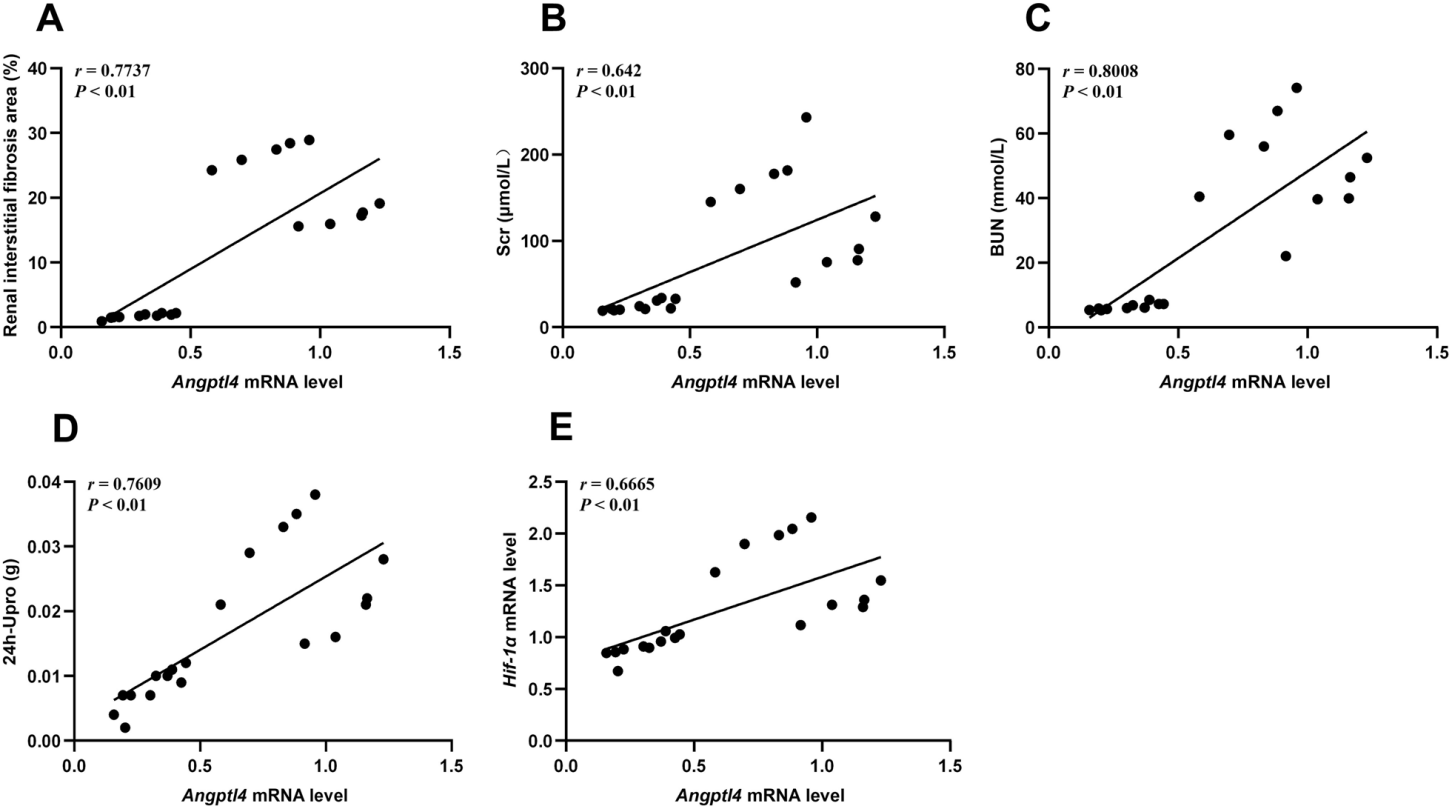
Correlation analysis of *Angptl4* levels with renal injury markers and *Hif-1α* levels. **A** Analysis of the correlation between *Angptl4* levels and renal interstitial fibrosis area. **B**–**D** Correlation analysis of *Angptl4* levels with Scr, BUN, and 24 h-Upro levels. **E** Analysis of the correlation between *Angptl4* and *Hif-1α*

To further ascertain the relationship between *Angptl4* mRNA and renal interstitial fibrosis in CKD rats, we conducted *Pearson* correlation analysis and simple linear regression. The renal interstitial fibrosis area in CKD rats was significantly positively correlated with the expression of *Angptl4* mRNA (*r* = 0.7737, *P* < 0.01) (Fig. 3A). Additionally, the expression of *Angptl4* mRNA exhibited a robust correlation with renal injury markers, including Scr, BUN, and UP (*r* = 0.642, 0.8008, and 0.7609, respectively; *P* < 0.01) (Fig. 3B–D). Consequently, ANGPTL4 may play a critical role in the progression of RIF in CKD rats. Furthermore, the expression level of *Angptl4* mRNA was positively correlated with the mRNA expression level of *Hif-1α* (*r* = 0.6665, *P* < 0.01) (Fig. 3E), suggesting a potential association between ANGPTL4 and HIF-1α.

### CKD patients with RIF exhibit elevated HIF-1α and ANGPTL4 expression

**Fig. 4:**
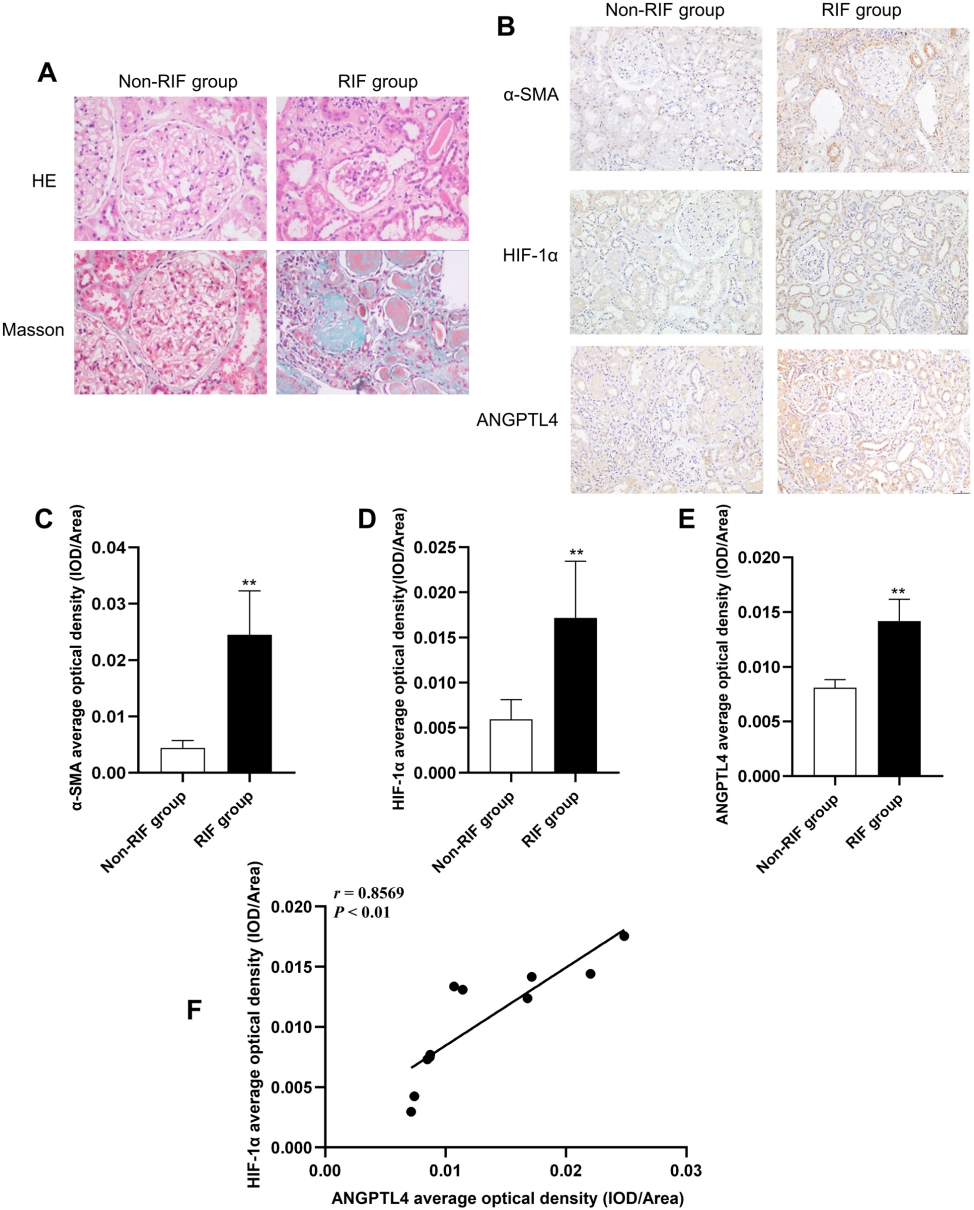
Increased expression of HIF-1α and ANGPTL4 in CKD patients with RIF. **A** Renal tissue was stained using HE and Masson's trichrome (× 400 magnification). **B** Immunohistochemical staining was used to assess the expression levels of HIF-1α, ANGPTL4, and α-SMA in the kidney (× 200 magnification, scale bar = 50 µm). **C**–**E** Quantification of immunohistochemical staining (the RIF group (n = 6) and the non-RIF group (n = 5)). **F** Analysis of the correlation between ANGPTL4 and HIF-1α. All the data are presented as the means ± standard deviations; *P < 0.05, **P < 0.01 vs. the non-RIF group

To validate the aforementioned findings in human samples, we analyzed the levels of HIF-1α and ANGPTL4 expression in renal biopsies from six CKD patients with RIF and five non-RIF CKD patients. The demographic and clinical information of the patients is detailed in Table 2. First, histological examinations using HE and Masson staining demonstrated varying degrees of blue collagen fiber deposition in the renal interstitium of the fibrosis group, accompanied by structural disarray in the kidney (Fig. 4A), in comparison to that in the nonfibrosis group. Then, immunohistochemical staining was conducted to observe the expression levels of HIF-1α and ANGPTL4. These results indicated a consistent increase in the expression of these genes, which was consistent with findings in rat models of adenine-induced RIF. Compared with those in the nonfibrosis group, the expression levels of HIF-1α and ANGPTL4 in the renal tissue of CKD patients with RIF were significantly elevated concomitant with the progression of RIF (Fig. 4B–E). Furthermore, *Pearson* correlation analysis revealed a significant positive correlation between the expression levels of ANGPTL4 and HIF-1α (*r* = 0.8569, *P* < 0.01) (Fig. 4F), confirming the association between ANGPTL4 and HIF-1α.

**Table 2:** Demographic and clinical information of the CKD patients

Group	Patient	Gender	Age	KDIGO	Nadir renal function		Proteinuria	Pathology of renal biopsy	
				CKD staging	sCr	eGFR		diagnosis	IFTA score
					(umol/l)	(ml/min/1.73m2)	(mg/24 h)		
RIF group	1	F	63	3	127.8	38.4	0.92	fpsIgAN	2
	2	M	48	3b	146.2	49.1	2.99	fpsIgAN	3
	3	M	51	3a	125.2	58	1.25	MsPGN	3
	4	F	48	2	192.2	26	1.01	MsPGN	2
	5	M	44	3b	180.7	39.1	0.69	FSGS	3
	6	M	71	3a	126.8	49.6	7.28	MN	2
Non-RIF group	1	M	16	1	81.2	125.1	0.77	MCN	0
	2	M	17	1	78.7	128.0	5.08	MCN	0
	3	M	31	1	85.5	105.9	9.24	MCN	0
	4	M	42	1	80.7	105.1	4.64	MCN	0
	5	M	29	1	82.4	112.3	2.39	MCN	0

M: male; F: female; fpsIgAN: focal proliferative sclerosing IgA nephropathy; MPGN: mesangial proliferative glomerulonephritis; FSGS: focal segmental glomerular sclerosis; MN: membranous nephropathy; MCN: minimal change nephrosis

### The HIF-1α-ANGPTL4 pathway is activated in hypoxia-induced HK2 cell fibrosis

**Fig. 5:**
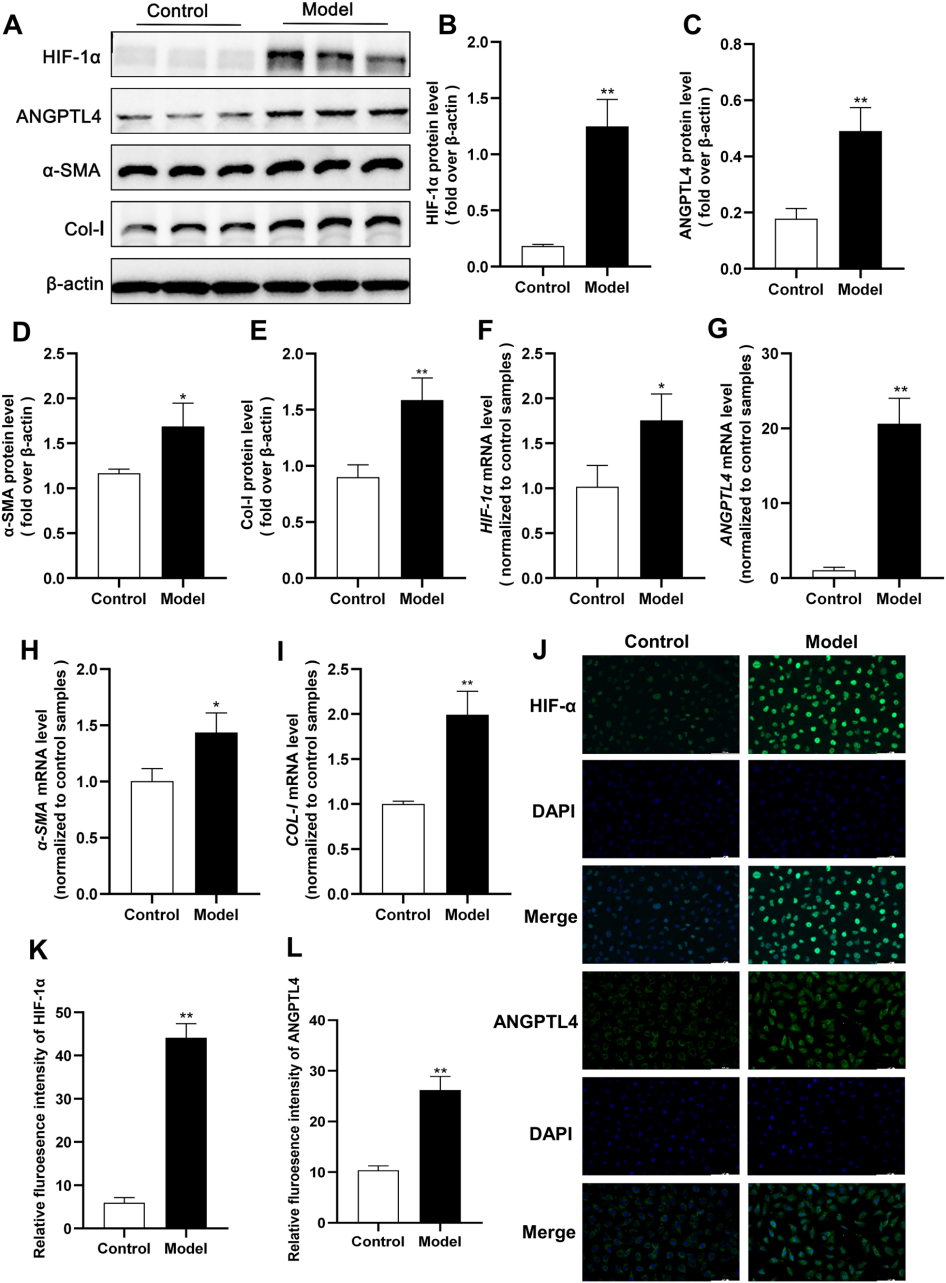
The HIF-1α-ANGPTL4 pathway is activated in hypoxia-induced HK2 cell fibrosis. After 24 h of hypoxia induction in HK2 cells, an in vivo model of RIF induction was established. **A** Western blot analysis was conducted to assess the protein levels of HIF-1α, ANGPTL4, α-SMA, and Col-I, and the protein levels were quantified using ImageJ software (n = 3/group) (**B**–**E**). **F**–**I** qRT‒PCR was used to measure the mRNA expression of *HIF-1α*, *ANGPTL4*, *α-SMA*, and *COL-I* in each group. **J**–**L** Immunofluorescence staining for HIF-1α and ANGPTL4 (× 200 magnification, scale bar = 100 µm). All the data are presented as the mean ± standard deviation. *P < 0.05, **P < 0.01 vs. the control group

**Fig. 6:**
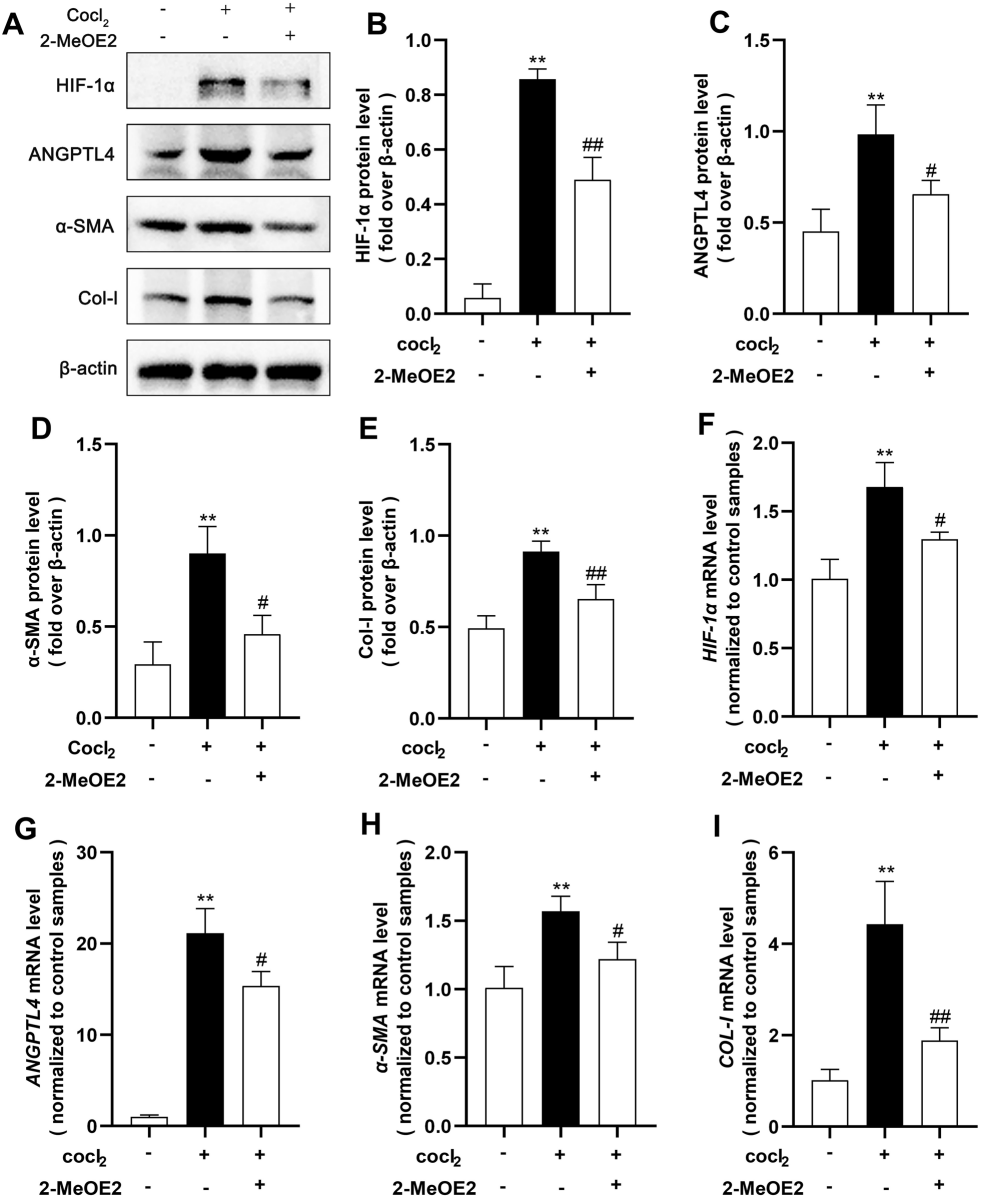
Impact of HIF-1α inhibitors on the levels of ANGPTL4 and fibrosis. HK2 cells were subjected to 2-MeOE2 (5 μmol/L) pretreatment for 12 h, followed by 24 h of hypoxia. **A** Western blot analysis was used to assess the protein levels of HIF-1α, ANGPTL4, α-SMA, and Col-I, and the protein levels were quantified using ImageJ software (n = 3/group) (**B**–**E)**. **F**–**I** qRT‒PCR was utilized to evaluate the mRNA expression of *HIF-1α*, *ANGPTL4*, *α-SMA*, and *COL-I* in each group. All the data are presented as the mean ± standard deviation. *P < 0.05, **P < 0.01 vs. the control group; #*P* < 0.05, ##*P* < 0.01 vs. the model group

To validate the impact of the HIF-1α-ANGPTL4 pathway on RIF, we initially induced fibrosis in HK2 cells through 24 h of hypoxia. In comparison to those in the control group, the Western blotting results revealed substantial increases in the protein expression levels of HIF-1α, ANGPTL4, and the fibrotic markers α-SMA and Col-I after hypoxia treatment (Fig. 5A–E). Similar patterns were observed for mRNA expression (Fig. 5F–I). Immunofluorescence analysis indicated a significant increase in the nuclear expression of HIF-1α and the cytoplasmic expression of ANGPTL4 in hypoxia-induced HK2 cells (Fig. 5J). Subsequently, to explore whether HIF-1α regulates the expression of ANGPTL4 and TIF, we pretreated HK2 cells with the HIF-1α inhibitor 2-MeOE2 (5 μmol/L) for 12 h, followed by continued hypoxia for 24 h. The results demonstrated successful inhibition of HIF-1α expression after 2-MeOE2 treatment, which led to a marked decrease in both the protein and mRNA levels of ANGPTL4. This intervention also suppressed the hypoxia-induced upregulation of α-SMA and Col-I in HK2 cells (Fig. 6A–E). These findings suggest that the HIF-1α-ANGPTL4 pathway is activated in hypoxia-induced HK2 cell fibrosis.

### Reduced or overexpressed ANGPTL4 affects HK2 cell fibrosis

**Fig. 7:**
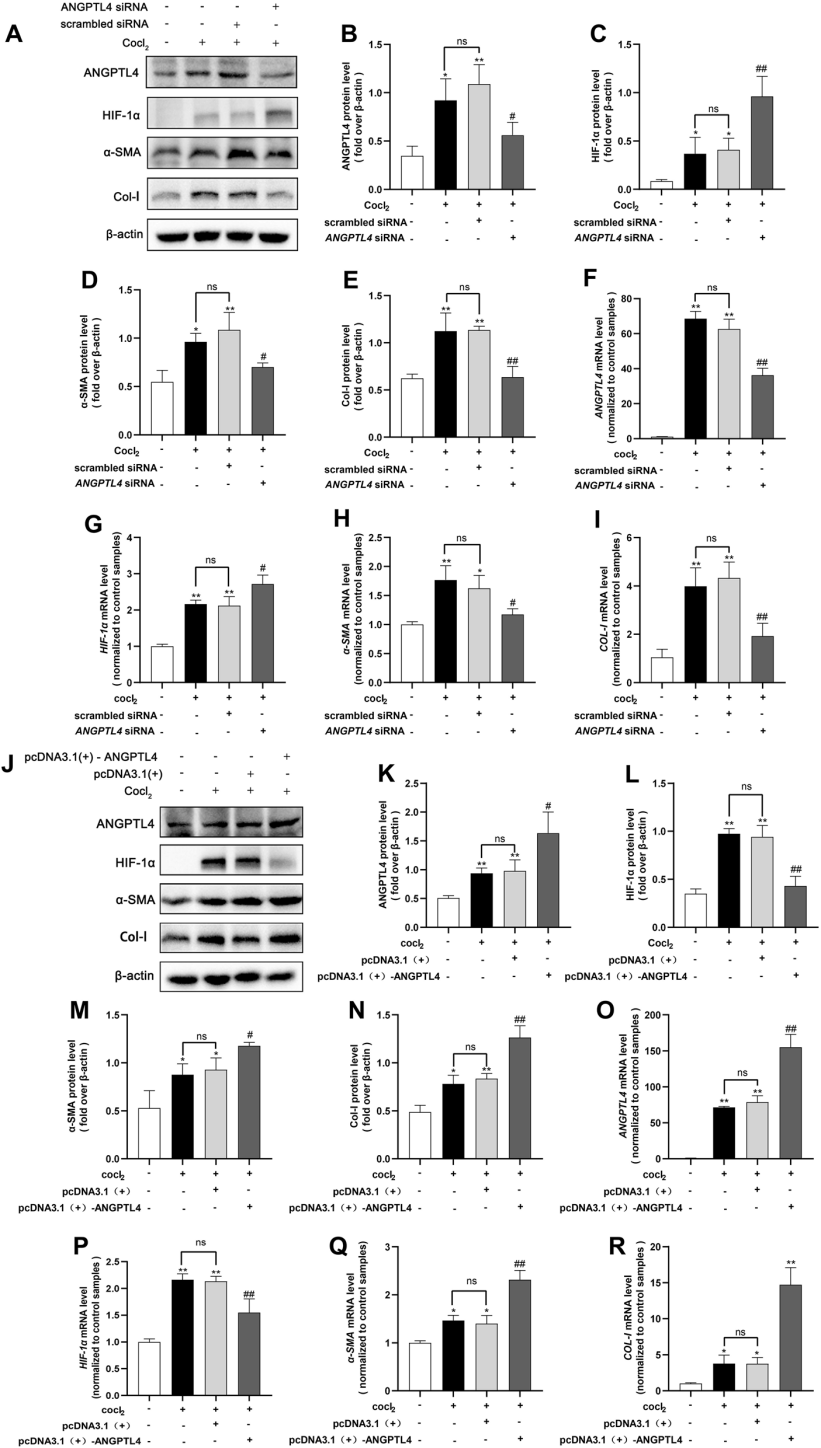
Reducing or overexpressing ANGPTL4 affects HK2 cell fibrosis. HK2 cells were transfected with *ANGPTL4* siRNA for 24 h, followed by hypoxia-induced fibrosis for an additional 24 h. **A** Western blotting analysis was conducted to assess the protein levels of ANGPTL4, HIF-1α, α-SMA, and Col-I, and the protein levels were quantified using ImageJ software (n = 3/group) (**B**–**E**). **F**–**I** qRT‒PCR was used to evaluate the mRNA levels of *ANGPTL4*, *HIF-1α*, *α-SMA*, and *COL-I* in each group. HK2 cells were transfected with the ANGPTL4 plasmid for 48 h. **J** Western blotting analysis was used to evaluate the protein levels of ANGPTL4, HIF-1α, α-SMA, and Col-I, and quantitative analysis was carried out using ImageJ software (**K**–**N**). **O**–**R** qRT‒PCR was utilized to examine the mRNA expression of *ANGPTL4*, *HIF-1α*, *α-SMA*, and *COL-I* in each group. All the data are presented as the mean ± standard deviation. *P < 0.05, **P < 0.01 compared to the control group; ^#^*P* < 0.05, ^##^*P* < 0.01 compared to the scrambled siRNA ( +) or pcDNA3.1 ( +) group; ns: not significant

To determine whether ANGPTL4 is a key factor in RIF, we transiently transfected HK2 cells with *ANGPTL4* siRNA or plasmids for 24 h, induced ANGPTL4 inhibition or overexpression, and subjected them to subsequent 24-h hypoxic exposure. Western blotting revealed that ANGPTL4 protein expression was significantly lower in the *ANGPTL4* siRNA group 48 h after transfection than in the scrambled siRNA group. Moreover, ANGPTL4 downregulation resulted in elevated levels of HIF-1α but strongly decreased the levels of the hypoxia-induced EMT-related marker α-SMA and the ECM deposition-related marker Col-I (Fig. 7A–E). Consistent results were demonstrated by qRT‒PCR analysis (Fig. 7F‒I). Similarly, overexpression of ANGPTL4 was evident in the pcDNA3.1( +)-ANGPTL4 group at 48 h posttransfection, in contrast to that in the pcDNA3.1( +) group. Upregulation of ANGPTL4 inhibited the expression of HIF-1α but exacerbated the increase in a-SMA and Col-I expression in hypoxia-induced HK2 cells (Fig. 7J–R). These results indicate that the expression level of ANGPTL4 can impact HK2 fibrosis, establishing ANGPTL4 as a crucial regulatory factor in RIF.

## Discussion

**Fig. 8:**
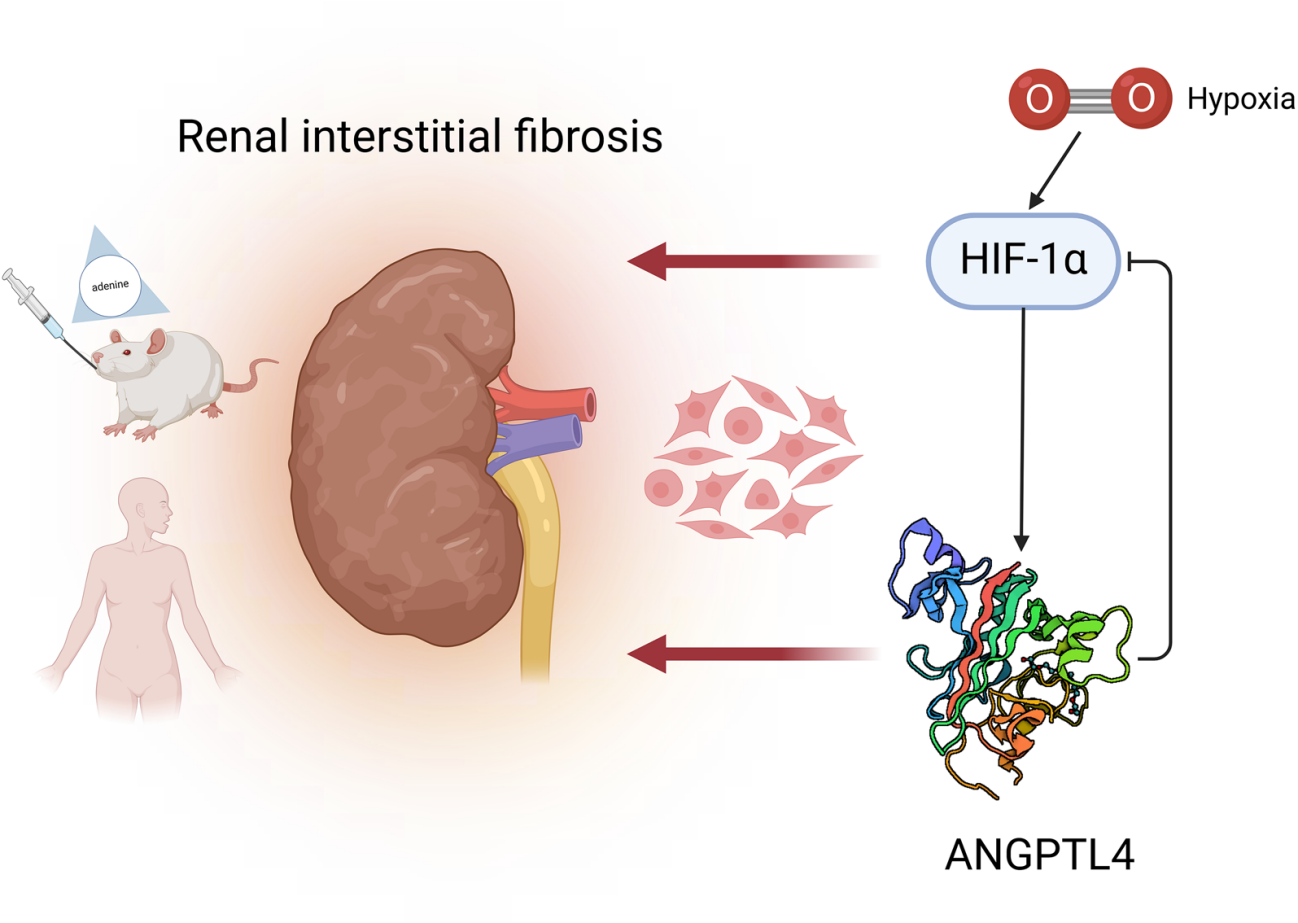
The model illustrates the role of ANGPTL4 in renal interstitial fibrosis. When renal interstitial fibrosis occurs due to various etiologies, the expression levels of HIF-1α and ANGPTL4 in the kidney significantly increase, and a negative regulatory mechanism is formed between the two. Modulating the expression of ANGPTL4 can markedly affect the accumulation of EMT and ECM components, ultimately impacting renal interstitial fibrosis. ANGPTL4 may serve as a pivotal molecular player in the occurrence and progression of renal interstitial fibrosis and is potentially emerging as a novel therapeutic target. Created with BioRender.com

In this study, we established an adenine-induced CKD rat model and a hypoxia-induced HK2 cell fibrosis model. A notable increase in the expression of ANGPTL4 was detected in both fibrotic kidneys and cells, and its preliminary validation was conducted on clinical samples. Building upon this foundation, we postulated that ANGPTL4 may influence the progression of RIF. Its impact on the progression of renal fibrosis was examined through the modulation of ANGPTL4 expression levels in vitro. Ultimately, ANGPTL4 was confirmed to be a critical molecular player in the occurrence and development of RIF in CKD, and it interacts with HIF-1α to form a regulatory loop (Fig. 8).

ANGPTL4 is highly expressed in the adipose tissue and liver of both humans and mice, while its expression in the heart, muscles, kidneys, skin, and other tissues is comparatively reduced [24, 25]. Its expression is subject to regulation by nutritional factors such as fasting; diverse metabolic states, including hypoxia; and inflammatory conditions [26, 27]. Rademaker et al. [28], utilizing a sheep model, investigated acute kidney injury induced by acute decompensated heart failure. Transcriptome analysis revealed that ANGPTL4 might serve as a potential candidate marker for long-term renal dysfunction triggered by acute decompensated heart failure. It is also considered a diagnostic marker for both primary and metastatic clear-cell renal cell carcinoma [29]. Recent studies have reported that ANGPTL4 is upregulated in models of conditions such as IgA nephropathy [30], cisplatin-induced acute kidney injury [31], nephrotic syndrome [32–34], and diabetic nephropathy [35–37], promoting renal injury. To examine the expression pattern of ANGPTL4 in RIF, we established a CKD rat model through the intragastric administration of low-dose adenine. This effect was primarily achieved by adenine-induced persistent renal damage, glomerulosclerosis, tubular atrophy, and interstitial fibrosis, a recognized model closely resembling the gradual progression of human CKD [38]. This study employed this method to establish a model. It is clear that the CKD group showed a decrease in renal function in comparison to the control group, along with a significant increase in 24-h urinary protein excretion. Histological examinations utilizing HE and Masson staining revealed renal structural disarray and substantial collagen fiber deposition in the CKD group. The protein and mRNA levels of α-SMA and Col-I markedly increased and progressively increased with time, confirming the successful establishment of the model. In our study, the protein and mRNA expression levels of ANGPTL4 at various time points in the CKD group were significantly greater than those in the control group and showed a significant positive correlation with renal injury markers. Moreover, a marked increase in ANGPTL4 was also observed in the kidneys of CKD patients with RIF. These findings imply the potential involvement of ANGPTL4 in the occurrence and development of CKD-related RIF, which warrants further exploration.

HIF-1α can induce RIF through the modulation of gene transcription, cross-talk with various signaling pathways, EMT, and epigenetic regulation [14]. Furthermore, the regulation of ANGPTL4 expression by HIF-1α has been confirmed in various diseases, such as tumors, diabetes, and arterial sclerosis resulting from chronic intermittent hypoxia [13, 39, 40]. Nonetheless, research on this phenomenon in the context of the kidneys is limited. Liu et al.'s study [10] revealed a significant reduction in ANGPTL4 expression in a model of aristolochic acid nephropathy following the administration of a specific HIF-1α inhibitor. In our study, elevated HIF-1α protein and mRNA expression was distinctly observed in the fibrotic kidneys of CKD rats and patients compared to those of the control group, and the expression levels of HIF-1α were significantly positively correlated with ANGPTL4. Subsequent in vitro experiments induced fibrosis in HK2 cells through hypoxia, reaffirming the upregulation of HIF-1α and ANGPTL4 expression in the RIF model. After HK2 cells were treated with a HIF-1α inhibitor, the expression of ANGPTL4 significantly decreased. However, knocking down or overexpressing ANGPTL4 in turn affected the expression of HIF-1α. These findings indicate that HIF-1α can regulate the expression of ANGPTL4, which in turn is subject to negative feedback regulation by ANGPTL4, leading to a balanced state between the two. These findings reveal the presence of a negative feedback regulatory mechanism between HIF-1α and ANGPTL4 in RIF.

Recently, the role of ANGPTL4 in fibrosis has remained a subject of interest. Saito et al. [19] reported that in patients with idiopathic pulmonary fibrosis and in a bleomycin-induced mouse model of pulmonary fibrosis, the expression of ANGPTL4 was significantly upregulated. Additionally, they used a recombinant ANGPTL4 intervention, which made the progression of pulmonary fibrosis much worse. In contrast, mice lacking ANGPTL4 showed improvements. Additionally, ANGPTL4 has been shown to be significantly upregulated in patients with cirrhosis and in cellular models of liver injury induced by the hepatitis C virus. Interfering with ANGPTL4 through the TLR4/NF-κB pathway can inhibit fibrosis in hepatic stellate cells and impede the progression of liver cirrhosis in mice [17, 41]. However, there is currently a lack of pertinent research on whether ANGPTL4 influences renal fibrosis. To further explore the impact of ANGPTL4 on RIF, we altered the expression of ANGPTL4 by transfecting HK2 cells with ANGPTL4 siRNA and plasmids. Our study revealed that the knockdown or overexpression of ANGPTL4 significantly suppressed or promoted the expression of the fibrosis markers α-SMA and Col-I, respectively. Therefore, our research revealed that ANGPTL4 may exacerbate the progression of RIF. Notably, following ANGPTL4 knockdown, the level of α-SMA remained greater than that in the control group, indicating that ANGPTL4 may not be the sole regulatory factor in renal fibrosis and that other HIF-1α-mediated regulatory pathways, such as the PI3K/AKT signaling pathway, may also be involved [42]. Paradoxically, research has reported divergent effects of ANGPTL4 on cardiac fibrosis [43]. ANGPTL4 has been shown to play a potential protective role in a mouse model of angiotensin II-induced atrial fibrosis and in cardiac fibroblasts by modulating signaling pathways such as the PPARγ pathway [44, 45]. Furthermore, in a nonalcoholic fatty liver disease model, the absence of ANGPTL4 leads to the accumulation of free cholesterol in hepatic stellate cells, thereby exacerbating liver fibrosis [46]. These studies suggest that different intervention factors can induce varied expression levels of ANGPTL4 in different cell types, possibly attributed to the integrated functionality of its unique N-terminal and C-terminal domains. The specific outcomes depend on how the protein is hydrolyzed, cleaved, or changed after translation, leading to distinctly divergent pathological or therapeutic effects.

Nevertheless, there are several limitations to this investigation. First, the adenine-induced CKD rat model might not be representative of all forms of renal fibrosis. Second, we did not delve into the downstream mechanisms through which ANGPTL4 influences RIF. Finally, although we treated cells with HIF-1α inhibitors and knocked down or overexpressed ANGPTL4 in vitro, there is still a lack of in vivo studies on the use of HIF-1α and ANGPTL4 inhibitors in RIF, and future experiments in multiple renal models are needed for validation. Therefore, these aspects will be focal points in our forthcoming research endeavors.

## Conclusions

This research substantiates, for the first time, that ANGPTL4 serves as a pivotal regulatory factor in RIF. Our study revealed the activation of the ANGPTL4 signaling pathway in both CKD rat models and patients. Furthermore, the interaction between ANGPTL4 and HIF-1α forms a regulatory loop, thereby modulating fibrosis-associated markers and actively participating in the progression of RIF. These findings suggest that ANGPTL4 may be a novel therapeutic target for the prevention and treatment of RIF.

## Data Availability

All the data generated or analyzed during this research period were incorporated into the published manuscript.

## Abbreviations

24 h-Upro: 24-Hour urine protein
ANGPTL4: Angiopoietin-like 4
BUN: Blood urea nitrogen
CKD: Chronic kidney disease
Col-I: Collagen I
ECM: Extracellular matrix
EMT: Epithelial mesenchymal transition
HIF-1α: Hypoxia-inducible factor-1α
qRT‒PCR: Quantitative real-time polymerase chain reaction
RIF: Renal interstitial fibrosis
Scr: Serum creatinine
siRNA: Small interfering RNA
α-SMA: α-Smooth muscle actin
