# Correction: The ANGPTL4-HIF-1α loop: a critical regulator of renal interstitial fibrosis

**DOI:** 10.1186/s12967-024-05586-w

**Published:** 2024-08-16

**Authors:** Yan Li, Shuang Chen, Qian Yang, Xiao Liu, Weiming Zhou, Ting Kang, Weihua Wu, Santao Ou

**Affiliations:** 1 Department of Nephrology, The Affiliated Hospital of Southwest Medical University, 25 Taiping Street, Jiangyang District, Luzhou, 646000 Sichuan China; 2 Sichuan Clinical Research Center for Nephrology, Luzhou, 646000 Sichuan China; 3 Metabolic Vascular Disease Key Laboratory of Sichuan Province, Luzhou, 646000 Sichuan China

**Correction: Journal of Translational Medicine (2024) 22:649** 10.1186/s12967-024-05466-3

Following publication of the original article [1], we have been notified that Fig. 2 was published incorrectly.

It is now:
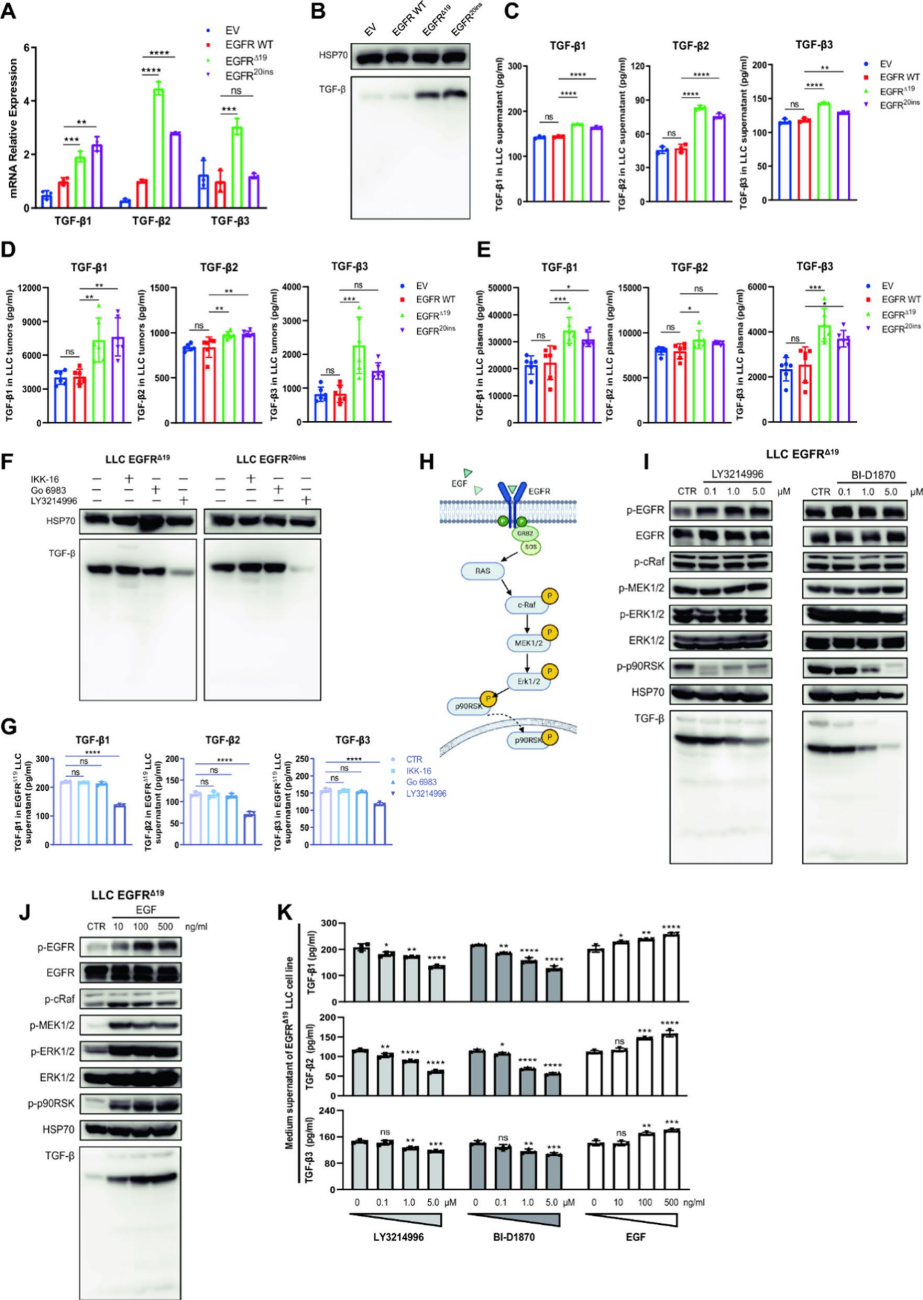


It should be:

**Fig. 2:**
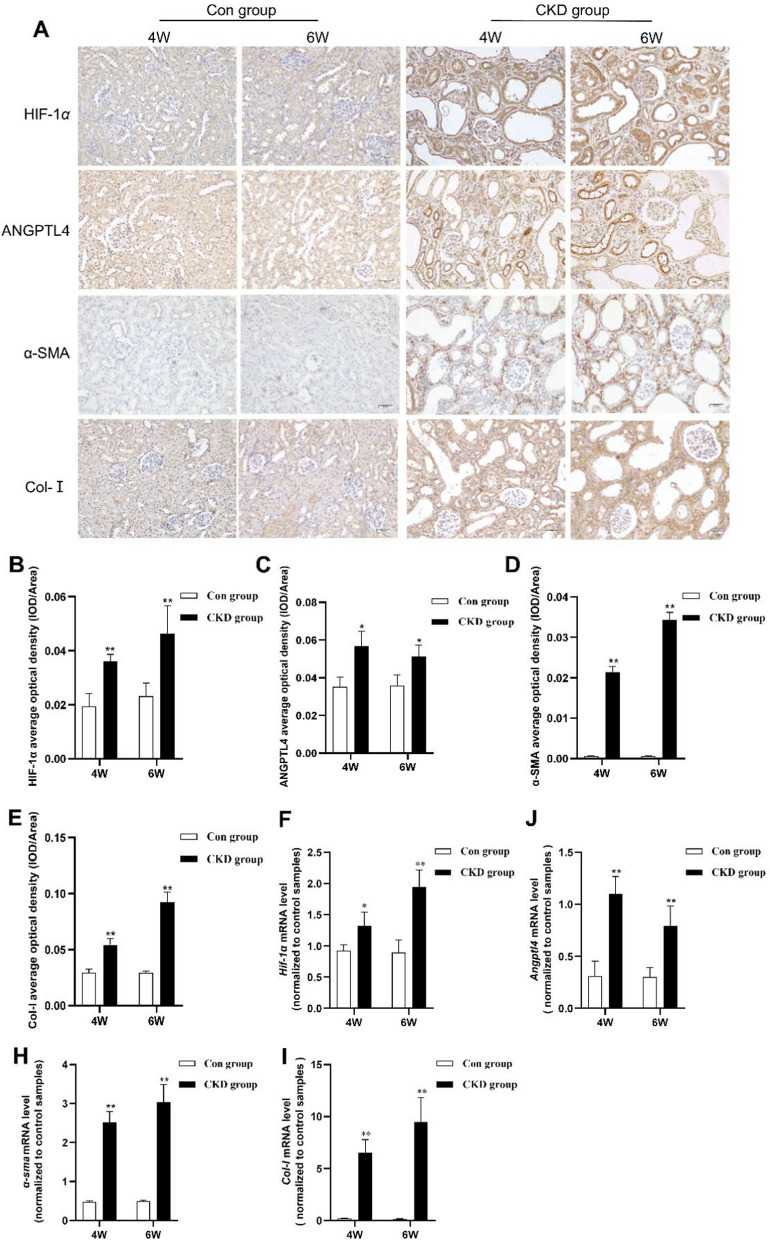
Increased expression of HIF-1α and ANGPTL4 in CKD rats. **A** Immunohistochemical staining to assess the expression of HIF-1α, ANGPTL4, α-SMA, and Col-I in the renal tissues of the two groups (× 200 magnification, scale bar = 50 µm). **B**–**E** Quantification of immunohistochemical staining (n = 5/group). **F**–**I** qRT‒PCR analysis of the expression of *Hif-1α*, *Angptl4*, *α-sma*, and *Col-I* mRNA in the renal tissues of the two groups of rats. All the data are presented as the mean ± standard deviation; *P < 0.05, **P < 0.01 vs. the corresponding control group at the same time point

The original article [1] was updated.
